# Risk factors for postoperative acute ischemic stroke in advanced-aged patients with previous stroke undergoing noncardiac surgery: a retrospective cohort study

**DOI:** 10.1186/s12893-023-02162-9

**Published:** 2023-08-29

**Authors:** Wei Xiao, Shuyi Yang, Shuai Feng, Chunxiu Wang, Hao Huang, Chaodong Wang, Chonglin Zhong, Shubin Zhan, Dongxu Yao, Tianlong Wang

**Affiliations:** 1 Department of Anesthesiology, Xuanwu Hospital, Capital Medical University, Beijing, 100053 China; 2 Department of Evidence-based Medicine, Xuanwu Hospital, Capital Medical University, Beijing, China; 3 Department of Medical Records and Statistics, Xuanwu Hospital, Capital Medical University, Beijing, China; 4 Department of Neurology, Xuanwu Hospital, National Clinical Research Center for Geriatric Diseases, Capital Medical University, Beijing, China

**Keywords:** Advanced-aged patients, Previous stroke, Noncardiac surgery, Postoperative ischemic stroke, Risk factors

## Abstract

**Background:**

The current study aimed to investigate the incidence and risk factors for postoperative acute ischemic stroke (PAIS) in advanced-aged patients (≥ 75 years) with previous ischemic stroke undergoing noncardiac surgery.

**Methods:**

In this single-center retrospective cohort study, all advanced-aged patients underwent noncardiac surgery from 1 January, 2019, to 30 April, 2022. Data were extracted from hospital electronic medical records. Multivariable logistic regression analysis was performed to determine predictors of PAIS. Multivariable linear or logistic regression analysis was performed to determine predictors of outcomes due to PAIS.

**Results:**

Twenty-four patients (6.0%) of the 400 patients developed PAIS. Carotid endarterectomy (CEA), length of surgery and preoperative Modified Rankin scale (mRS) ≥ 3 were significant predictors of PAIS. CEA was associated with increased risk of PAIS (OR 4.14; 95%CI, 1.43–11.99). Each additional minute in length of surgery had slightly increased the risk of PAIS (OR, 1.01; 95%CI, 1.00-1.01). Compared with reference (mRS < 3), mRS ≥ 3 increased odds of PAIS (OR, 4.09;95%CI, 1.12–14.93). Surgery type and length of surgery were found to be significant predictors of in-hospital expense (*P <* 0.001) and hospital stays (*P <* 0.05).

**Conclusions:**

CEA, length of surgery and preoperative mRS ≥ 3 may increase the development of PAIS in advanced-aged patients (≥ 75 years) with previous stroke undergoing noncardiac surgery. PAIS increased in-hospital mortality and prolonged hospital stay.

**Supplementary Information:**

The online version contains supplementary material available at 10.1186/s12893-023-02162-9.

## Introduction

Postoperative stroke is a well-recognized complication following surgery. The reported risk of postoperative stroke varies with surgery types [1]. The incidence of postoperative stroke in elderly patients undergoing noncardiac surgery has been reported to range from 0.3 to 7% [2, 3]. Postoperative stroke can prolong hospital stay and increase mortality.

Cerebrovascular reserve is impaired because of the increased burden of atherosclerosis with age. An age of ≥ 75 years was found to be an independent risk factor for cerebral vascular event following elective orthopedic procedures [4]. Previous stroke also markedly raised the risk of postoperative stroke [5]. Given the rapidly rising trend of the aged population undergoing surgery, advanced-aged patients with previous stroke may lead to a higher incidence of postoperative stroke.

The research question of this study was what were the risk factors of PAIS in advanced-aged patients (≥ 75 years) with previous stroke undergoing noncardiac surgery.

## Materials and methods

### Study design

This study was designed as an observational study in a retrospective cohort of advanced-aged patients (≥ 75 years) with previous ischemic stroke undergoing noncardiac procedures. The current study was fully approved by the ethics committee of Xuanwu Hospital, Capital Medical University (Lin Yan Shen [2018] No.086). No written informed consent was required due to the observational nature of the study.

### Study population

**Fig. 1:**
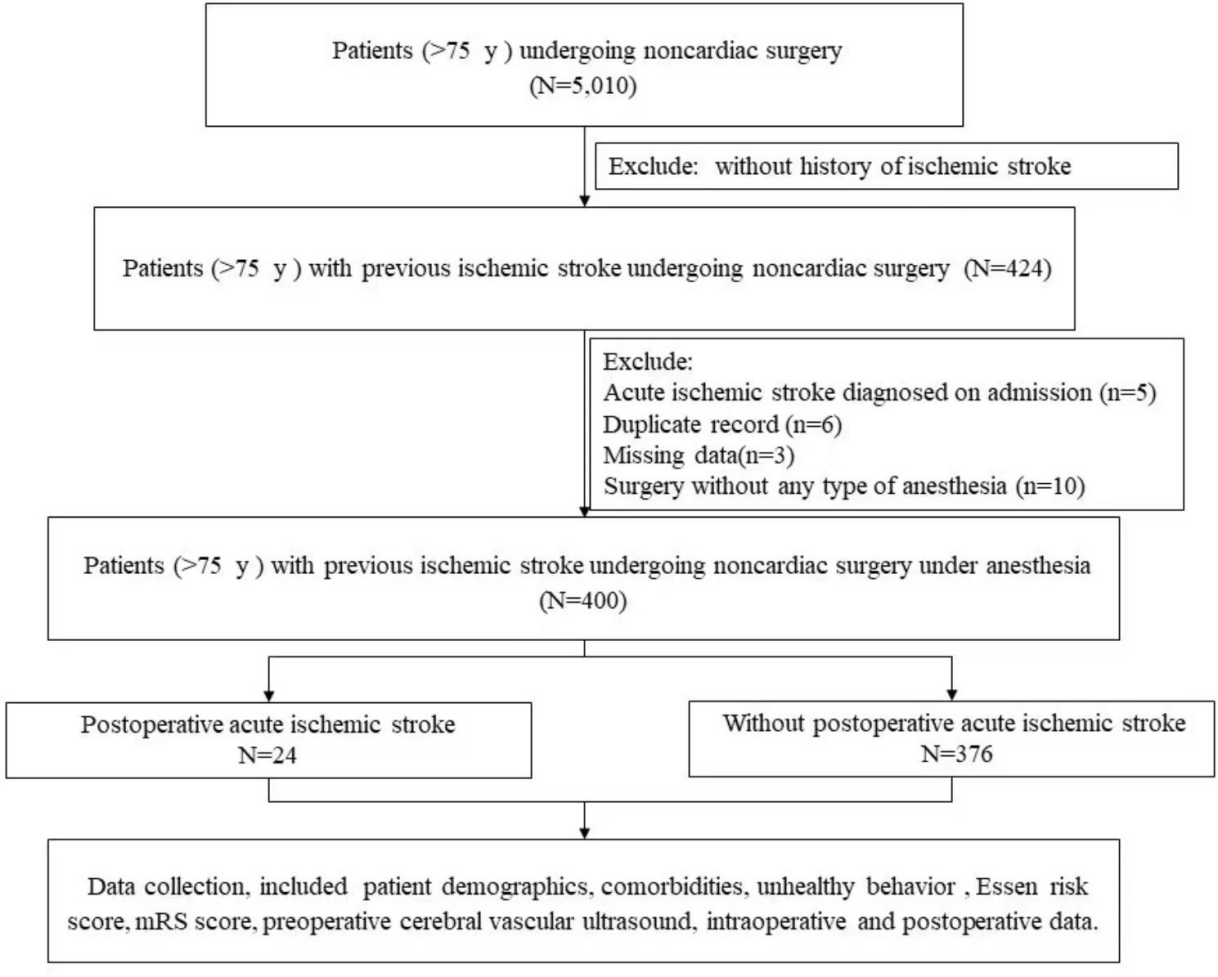
Study flowchart. mRS, modified rankin scale

This study included consecutive advanced-aged patients (≥ 75 years) with history of ischemic stroke. All patients underwent noncardiac surgeries from 1 January, 2019, to 30 April, 2022 in Xuanwu Hospital. The following were the exclusion criteria: acute ischemic stroke diagnosed on admission, duplicate record, missing data, surgery without any type of anesthesia (Fig. 1). Patients with acute ischemic stroke diagnosed on admission required urgent treatment for their stroke, these patients were consequently excluded. The ischemic stroke was defined as an episode of neurological dysfunction caused by focal cerebral, spinal, or retinal infarction [6]. Covert ischemic stroke, which defined as an acute infarction on brain magnetic resonance imaging (MRI) without clinical diagnosis of stroke before the MRI, was also counted as ischemic stroke [7]. Considering the availability of medical records, postoperative ischemic stroke was defined as an ischemic stroke occurred during the postoperative hospitalization period in our study. The PAIS was diagnosed according to the medical record and brain MRI together, and the diagnosis of PAIS was further validated by a consultant neurologist who was blinded to the study design.

### Data collection

All demographic data and comorbidities were collected from electronic medical records. Demographic data included age, sex and American Society of Anesthesiologists (ASA) classification. Comorbidities included the following diseases: tobacco use, congestive heart failure (CHF), myocardial infarction (MI), atrial fibrillation (AF), other cardiovascular disease (except MI and AF), hypertension, transient ischemic attack (TIA), peripheral vascular disease, hyperlipidemia and diabetes mellitus (DM). MI was defined as a myocardial infarction occurring within 6 months preceding surgery. DM was classified by insulin dependent. Essen risk score [3] was used to assess recurrent risk of ischemic stroke. The Essen score contains 8 items: age, history of hypertension, history of diabetes mellitus, history of MI, other cardiovascular disease except for MI and atrial fibrillation, history of peripheral arterial disease, ever smoking and previous ischemic stroke or TIA. The Essen score ranges from 0 to 9. Considering all patients enrolled in this study were ≥ 75 years (scoring 2 points) and with previous ischemic stroke (scoring 1 point), their Essen risk scores were thus at least 3 points. In Essen risk scoring system, 3 points or above 6 points respectively mean a one-year recurrent rate for stroke at 4-7% or 7-11% [3]. Essen risk score was therefore categorized at the boundary score of 7 in this study. Modified Rankin scale (mRS) score was used to classify the degree of preoperative disability. The mRS score was an ordered scale from 0 to 6 [8].As moderate disability was defined as 3 or above [8], this score was used as cut-off in this study. Cerebral vascular stenosis was defined as more than 50% stenosis in cerebral vascular detected by preoperative vascular ultrasound [9].Use of antiplatelet drugs were also recorded. All anticoagulation agents were stopped before surgery according to the guidelines [10, 11].

Intraoperative data were extracted from the anesthesia recording system. Blood pressure (BP) was stored every 5 min in this system. The following intraoperative data were extracted: urgency of surgery, surgery type, anesthesia type, length of surgery, length of anesthesia, transfusion, fluid management strategy, blood loss, BP variation, regional cerebral oxygen saturation(rScO_2_) monitoring, bispectral index (BIS) monitoring, use of ulinastatin, use of anti-fibrinolytic (tranexamic acid) and use of hemostatic (hemocoagulase) drugs. Ulinastatin was used for anti-inflammation, and the use of antifibrinolytic or hemostatic drugs were dependent on surgeons’ decision to reduce blood loss. Surgery type was categorized as “non-vascular surgery”, “peripheral vascular surgery” or “carotid endarterectomy (CEA)”. Non-vascular surgery included general surgery (gastrointestinal surgery, biliary surgery and thyroid surgery), neurosurgery (cerebral tumor resection, functional neurosurgery and ventriculoperitoneal shunt), orthopedic surgery (arthroplasty, spinal surgery and internal fixation of fracture), thoracic surgery (lung and esophageal surgery), gynecologic surgery (ovarian and uterine surgery) and urologic surgery (prostate surgery, bladder surgery and ureteral surgery). Anesthesia type was classified as either “general anesthesia” or not. Intraoperative BP variation was categorized according to the maximum BP changes during the whole procedure, which lasted at least 5 min. The baseline BP was defined as the mean of BP measured in the preoperative ward and all available BP measurements in the operating room before anesthesia induction [12]. Intraoperative BP variation was classified as “<10% of baseline value”, “10%-20% of baseline value” or “>20% of baseline value”.

Postoperative data were collected from the hospital medical records. The following postoperative data were included: in-hospital death, in-hospital expense, length of hospital stay (LOS), and laboratory blood tests (hemoglobin, platelet count and creatinine) on postoperative day one. In-hospital death was all -cause death before discharge. Postoperative hemoglobin level was categorized as reported [13]. Platelet count and creatinine were both classified dependent on their normal limits. The normal platelet count was between 100 × 10^9^ and 300 × 10^9^.The normal limit of creatinine was below 104 µmol/L.

### Statistical analysis

SPSS 26.0 statistical software was used for statistical analyses (SPSS, IBM, USA). The normality of a continuous variable distribution was assessed by the Kolmogorov Smirnov test or Shapiro-Wilk test. Most perioperative characteristics were dichotomized or trichotomized at the clinical reference value, and their frequencies were calculated.

In the univariable analysis, differences in frequency of perioperative characteristics were identified by the Pearson chi-square test or the Fisher exact test where appropriate. Differences in continuous perioperative characteristics were identified by Student *t* test or the Mann-Whitney U test dependent on their normal distribution.

Surgery type was already suggested as a risk factor according to previous literature [1]. Except for the outcome variables (in-hospital death, length of hospital stays and in-hospital expense) and interrelated variables, “surgery type” and all other individual variables significant with nominal two-tailed *P* value < 0.05 in the univariable analysis thus entered into multivariable logistic or linear regression models. In the univariable analysis, *P* values were adjusted with Bonferroni correction, otherwise a *P* value < 0.05 was considered statistical significance.

## Results

During the study period, 5,010 advanced-aged patients underwent noncardiac surgery. A total of 424 advanced-aged patients with previous ischemic stroke was enrolled into this study. 24 patients were excluded due to acute ischemic stroke on admission (n = 5), duplicate record (n = 6), missing data (n = 3) and surgery without any type of anesthesia (n = 10). Of the 400 patients finally included into analysis, 202 (50.5%) were men. The age range was 85–97 years, and the median (interquartile range) age was 78.0 (76.0–86.0) years. After the medical electronic records and original MRIs were reviewed by the consultant neurologist, the diagnosis of PAIS was confirmed in 24 patients (6.0%). Covert stroke was diagnosed in 16 patients (4.0%). The follow up duration was before discharge. The incidence of PAIS in patients undergoing CEA was 13.7%. As a long-term surgery was defined as a length of surgery ≥ 3 h [8], which was used as cut-off in this study. The incidence of PAIS was significantly higher in patients with length of surgery ≥ 3 h (13.9% vs. 3.1%, *P* < 0.001, Supplemental Table 1) and preoperative mRS ≥ 3 (16.1% vs. 5.1%, *P* = 0.030, Supplemental Table 1). Study flowchart was shown in Fig. 1.

### Outcomes due to PAIS

Patients with PAIS had a higher incidence of in-hospital death (8.3% vs. 0.5%, *P* < 0.001) and longer length of hospital stay (14.0 (7.0–16.0) d vs. 8.0 (6.0–12.0) d, *P* = 0.020) than those without PAIS. In-hospital expense was likely higher in patients with PAIS than those without PAIS (97077.95 (51310.45-154616.93) yuan vs. 68475.48 (44635.63-101518.68) yuan, *P* = 0.050) (Table 1).

**Table 1:** Patients’ Outcomes

	No stroke (n = 376)	Stroke (n = 24)	*P-*value
In-hospital expense, yuan	68475.48 (44635.63-101518.68)	97077.95 (51310.45-154616.93)	0.050
In-hospital death (%)	1 (0.5)	2 (8.3)	< 0.001
LOS, d	8.0 (6.0–12.0)	14.0 (7.0–16.0)	0.020

Note: Values are expressed as percentages or median (interquartile)

## Preoperative characteristics and PAIS

After adjusted with Bonferroni correction, no significant differences were observed according to sex, age, tobacco use, hyperlipidemia, high ASA classification (≥ III), high Essen risk score (≥ 7), high preoperative mRS scores (≥ 3), cerebral vascular stenosis or usage of antiplatelet drugs between the two groups. History of CHF, AF, MI, hypertension, other cardiovascular disease, TIA, peripheral vascular disease or DM was not significantly different between patients with PAIS and those without PAIS. (Table 2).

**Table 2:** Univariate Analysis of Preoperative Characteristics and PAIS

	No stroke (n = 376)	Stroke (n = 24)	*P*-value
Sex (Male, %)			0.273
No	200 (53.2)	10 (41.7)	
Yes	176 (46.8)	14 (58.3)	
Age, y	81.0 (77.0-86.3)	77.0 (76.0–80.0)	0.067
ASA classification ≥ III (%)			0.044
No	64 (17.0)	8 (33.3)	
Yes	312 (83.0)	16 (66.7)	
CHF history (%)			> 0.999
No	371 (98.7)	24 (100)	
Yes	5 (1.3)	0	
TIA history (%)			0.558
No	364 (96.8)	23 (95.8)	
Yes	12 (3.2)	1 (4.2)	
AF history (%)			0.890
No	358 (95.2)	23 (95.8)	
Yes	18 (4.8)	1 (4.2)	
Hyperlipidemia (%)			0.084
No	309 (82.2)	23 (95.8)	
Yes	67 (17.8)	1 (4.2)	
MI history^a^ (%)			> 0.999
No	366 (97.3)	24 (100)	
Yes	10 (2.7)	0	
Other cardiovascular disease history (%)			0.986
No	250 (66.5)	16 (66.7)	
Yes	126 (33.5)	8 (33.3)	
Peripheral vascular disease history (%)			> 0.999
No	365 (96.8)	24 (100)	
Yes	11 (3.2)	0	
Hypertension history (%)			0.359
No	108 (28.7)	9 (37.5)	
Yes	268 (71.3)	15 (62.5)	
Tobacco use (%)			0.108
No	302 (80.3)	16 (66.7)	
Yes	74 (19.7)	8 (33.3)	
DM history (%)			0.270
None	253 (67.3)	19 (79.2)	
Non-insulin dependent	91 (24.2)	5 (20.8)	
Insulin dependent	32 (8.5)	0	
Essen risk score ≥ 7 (%)			0.259
No	357 (95.0)	24 (100)	
Yes	19 (5.0)	0	
Preoperative mRS score ≥ 3 (%)			0.011
No	351 (93.4)	19 (79.2)	
Yes	25 (6.6)	5 (20.8)	
Cerebral vascular stenosis (%)			0.733
No	70 (18.7)	6 (25.0)	
Yes	181 (48.1)	11 (45.8)	
Unknown	125 (33.2)	7 (29.2)	
Antiplatelet drugs (%)			0.107
No	330 (87.8)	18 (75.0)	
Yes	46 (12.2)	6 (25.0)	

Note: Values are expressed as percentage or median (interquartile)

^a^ Defined as myocardial infarction occurring during the 6 months preceding surgery. CHF history, TIA history, MI history and peripheral vascular disease history were analyzed by Fisher exact test.

After adjusted with Bonferroni correction, *P* value < 0.003(0.003 = 0.05/numbers of univariates in Table 2 ) was considered statistical significance.

## Intraoperative characteristics and PAIS

After adjusted with Bonferroni correction, univariable analysis demonstrated that patients with PAIS had significantly longer length of surgery (*P* = 0.002) and anesthesia (*P* = 0.002). No significant differences were observed according to undergoing emergent surgery, undergoing CEA, general anesthesia, intraoperative transfusion, intraoperative negative volume load, blood loss or intraoperative BP variation between these two groups. There were also no obvious differences according to intraoperative use of BIS monitoring, rScO_2_ monitoring, ulinastatin, tranexamic acid or hemocoagulase between the two groups. (Table 3).

**Table 3:** Univariate Analysis of Intraoperative Characteristics and PAIS

	No stroke (n = 376)	Stroke (n = 24)	*P*-value
Emergent surgery (%)			0.350
No	360 (95.7)	22 (91.7)	
Yes	16 (4.3)	2 (8.3)	
Surgery type (%)			0.084
Non-vascular surgery	330 (87.8)	17 (70.8)	
Peripheral vascular surgery	1 (0.3)	0	
CEA	45 (11.9)	7 (29.2)	
General anesthesia (%)			0.014
No	76 (20.2)	0	
Yes	300 (79.8)	24 (100.0)	
Length of surgery, min	87.0 (59.5-144.3)	283.0 (88.0-305.0)	0.002
Length of anesthesia, min	161.0 (111.8-205.5)	329.00 (176.0-375.0)	0.002
Intraoperative transfusion (%)			0.363
No	265 (70.5)	19 (79.2)	
Yes	111 (29.5)	5 (20.8)	
Intraoperative negative volume load (%)			0.453
No	344 (91.5)	23 (95.8)	
Yes	32 (8.5)	1 (4.2)	
Blood loss, mL	50.0 (20.0-150.0)	100.0 (45.0-200.0)	0.096
Intraoperative BP variation (%)			0.462
< 10% of baseline	102 (27.1)	4 (16.7)	
10%-20% of baseline	139 (37.0)	9 (37.5)	
≥ 20% of baseline	135 (35.9)	11 (45.8)	
Intraoperative rScO_2_ monitoring (%)			1.000
No	329 (87.5)	21 (87.5)	
Yes	47 (12.5)	3 (12.5)	
Intraoperative BIS monitoring (%)			0.012
No	79 (21.01)	0	
Yes	297 (78.99)	24 (100.0)	
Intraoperative use of ulinastatin (%)			0.050
No	282 (75.0)	15 (62.5)	
Yes	94 (25.0)	9 (37.5)	
Intraoperative use of tranexamic acid (%)			0.873
No	341 (90.7)	22 (91.7)	
Yes	35 (9.3)	2 (8.3)	
Intraoperative use of hemocoagulase (%)			0.429
No	343 (91.2)	23 (95.8)	
Yes	33 (8.8)	1 (4.2)	

Values are expressed as percentage or median (interquartile)

After adjusted with Bonferroni correction, *P* value < 0.003(0.003 = 0.05/numbers of univariates in Table 3 ) was considered statistical significance

## Postoperative characteristics and PAIS

After adjusted with Bonferroni correction, univariate analysis indicated that no significant differences were revealed according to postoperative hemoglobin, platelet count or serum creatinine between patients with PAIS and those without PAIS (Table 4).

**Table 4:** Univariate Analysis of Postoperative Characteristics and PAIS

	No stroke (n = 376)	Stroke (n = 24)	*P-*value
Hemoglobin on postoperative day one (%)			0.502
≥ 9 g/dL	334 (91.5)	21 (87.5)	
< 9 g/dL	32 (8.5)	3 (12.5)	
Platelet count on postoperative day one (%)			0.592
< 100×10^9^/L	17 (4.5)	1 (4.2)	
100×10^9^~300×10^9^/L	333 (88.6)	20 (83.3)	
> 300×10^9^/L	26 (6.9)	3 (12.5)	
Serum Cr on postoperative day one (%)			0.950
≤ 104 µmol/L	346 (92.0)	22 (91.7)	
> 104 µmol/L	30 (8.0)	2 (8.3)	

Values are expressed as percentage

After adjusted with Bonferroni correction, *P* value < 0.017(0.017 = 0.05/numbers of univariates in Table 4 ) was considered statistical significance

## Multivariate Logistic/linear regression analysis of perioperative characteristics, PAIS and outcomes due to PAIS

Anesthesia type was correlated with surgery type (r = 0.196, *P* = 0.000), and BIS monitoring was routinely employed in patients under general anesthesia in our center. Length of anesthesia was related with surgery (r = 0.980, *P* = 0.000). Consequently, the above three interrelated variables were not included in the multivariable logistic or linear regression analysis.

Multivariate logistic regression analysis illustrated that CEA, length of surgery and preoperative mRS ≥ 3 were significant predictors of PAIS (R^2^ = 0.223, *P* < 0.05, Table 5), but not significant predictors of in-hospital death (*P* > 0.05) in advanced-aged patients with previous ischemic stroke undergoing noncardiac surgery. CEA was associated with increased risk of PAIS (OR, 4.14; 95%CI, 1.43–11.99; *P* = 0.009). Each additional minute in length of surgery had slightly increased the risk of PAIS (OR, 1.01; 95%CI, 1.00-1.01; *P* < 0.001). Compared with reference (mRS < 3), mRS ≥ 3 increased odds of PAIS (OR, 4.09;95%CI, 1.12–14.93; *P* = 0.033).

**Table 5:** Multivariate Logistic Analysis of Perioperative Characteristics and PAIS

Risk factors	N	Odd Ratios(95%CI)	*P*-value
Surgery-specific			
Surgery type			
Non-vascular surgery	348	1 (Ref.)	
Peripheral vascular surgery	1	0	> 0.999
CEA	51	4.14 (1.43–11.99)	0.009
Length of surgery, min		1.01 (1.00-1.01)	< 0.001
Patient-specific			
ASA			
ASA < 3	72	1 (Ref.)	
ASA ≥ 3	328	0.48 (0.18–1.31)	0.154
Preoperative mRS			
mRS < 3	370	1 (Ref.)	
mRS ≥ 3	30	4.09 (1.12–14.93)	0.033

Multivariate linear regression models for in-hospital expense (F = 43.275, adjusted R^2^ = 0.298, *P* < 0.001) and LOS (F = 10.927, adjusted R^2^ = 0.091, *P* < 0.001) were both significant. Surgery type and length of surgery were found to be significant predictors of in-hospital expense (*P <* 0.001, Supplemental Table 2). Similarly, both surgery type (*P* = 0.012, Supplemental Table 3) and length of surgery (*P <* 0.001, Supplemental Table 3) were significant predictors of LOS.

## Discussion

Advanced age and history of stroke were considered independent risk factors of postoperative stroke [14, 15]. Compared with younger control (< 50 years), risk of postoperative stroke markedly raised in patients older than 70 years undergoing noncardiac surgery [16]. In patients undergoing vascular surgery, each year of additional age was reported to slightly increase the risk of postoperative stroke (OR 1.02, 95%CI 1.01–1.04). The risk of postoperative stroke in patients with previous ischemic stroke was demonstrated at 2.5 to 3 times of those without stroke [17]. However, the incidence and risk factors of PAIS in advanced-aged patients with previous ischemic stroke undergoing noncardiac surgery are still unclear. We therefore performed a retrospective cohort study to demonstrate it. The incidence of PAIS was 6%. This study also revealed that CEA, length of surgery and preoperative mRS ≥ 3 were significant predictors of PAIS. Postoperative stroke obviously increased in-hospital mortality and prolonged hospital stay. Surgery type and length of surgery were shown as significant predictors of in-hospital expense and hospital stay.

Similar as our results, many studies found a strong association between CEA and postoperative stroke [18, 19]. As revealed in previous study, the incidence of postoperative stroke following CEA can range from 1.4 to 4% [20, 21]. Contributing factors of postoperative stroke in CEA included plaque emboli, platelet aggregates, improper flushing, poor cerebral protection and relative hypotension. Early postoperative stroke after CEA was reported to be an independent predictor for longer LOS, higher healthcare cost and worse long-term survival [8].

A prolonged surgery time was related with a greater increase in complications, postoperative hospital stay and 30-day mortality [20, 22, 23].Garcia et al. demonstrated that longer operative time was a strong predictor of postoperative stroke in patients with asymptomatic carotid stenosis undergoing revascularization [24]. Krafcik et al. also showed that re-operative CEA was associated with a longer operative time and higher risk of perioperative stroke [25]. Our result was consistent with literature.

The severity of previous stroke was assessed by mRS in our study. Initial neurologic condition indicated the perioperative stroke rate after CEA [26]. In patients undergoing early CEA with a nondisabling ischemic stroke, patients with a mRS ≥ 3 developed more postoperative neurologic worsening than those with a mRS ≤ 2 [26]. A mRS ≥ 3 was also identified as a significant risk factor of PAIS in our study, which was concordant with the previous finding.

ASA classification, an important indicator for co-morbidities, usually works as an anesthesia risk index. Yu et al. found an obvious correlation between high ASA classification and postoperative stroke occurrence in elderly patients undergoing hip fracture surgery [14]. In contrast to their study, we didn’t find a correlation between high ASA classification and PAIS. First potential reason is our cohort was already high-risk population for recurrent stroke, the contribution of high ASA level was therefore not easily reflected. Second possible reason is other predictors included in our model were with greater significances than ASA ≥ III. Future studies with a larger sample size are needed to test the predicting effect of high ASA level.

The Essen risk score was developed to predict the risk of recurrent stroke [3]. Weimar et al. revealed that one-year rate for recurrent stroke in the stable outpatient population of REACH (Reduction of Atherothrombosis for Continued Health) increased significantly in patients with Essen risk score from 0 to above 6 [3]. However, a high Essen risk score (≥ 7) was not found to be a significant predictor of PAIS in our study. Firstly, the patient cohort in our study was different. Our cohort, with at least 3 points on Essen risk score, was already high-risk population for recurrent stroke. Secondly, our primary outcome, which was in-hospital PAIS, also varied from Weimar et al’ s study.

Postoperative stroke has been reported to relate with increased morbidity and prolonged LOS [27, 28]. Postoperative stroke was associated with threefold to eightfold increases in 30-day mortality [27, 29]. Our results were consistent with literature.

However, our study has several limitations. Firstly, the current study was a single-institutional, retrospective study, which is prone to bias and confounding, the results of this study are consequently not representative of the general population. Secondly, the location and timing of the previous stroke before surgery and the timing of PAIS were not recorded, these may lead to bias. Thirdly, the “non-vascular surgery” group was heterogenous, which could confound findings. Fourthly, NIHSS scores of PAIS were not available due to the retrospective characteristics, the relationship between severity of stroke in patients with PAIS and surgical characteristics can’t be analyzed. Finally, the outcome variables of PAIS, such as discharge disposition, 30-day readmission and 30-day mortality could be expanded on in future studies.

## Conclusion

In summary, the incidence of PAIS was 6% in advanced- aged patients with previous ischemic stroke undergoing noncardiac surgery. CEA, length of surgery and preoperative mRS ≥ 3 might increase the development of PAIS. PAIS obviously increased in-hospital mortality and prolonged hospital stay. This knowledge helps to identify high-risk surgical patients with advanced age and previous ischemic stroke.

### Electronic supplementary material

Below is the link to the electronic supplementary material.

Additional File 1: Table s1-s3

## Data Availability

The datasets used and /or analyzed during the current study are available from the corresponding author on reasonable request.

## Abbreviations

PAIS: Postoperative acute ischemic stroke
LOS: Length of hospital stays
ASA: American Society of Anesthesiologists
CHF: Congestive heart failure
TIA: Transient ischemic attack
AF: Atrial fibrillation
MI: Myocardial infarction
DM: Diabetes mellitus
mRS: Modified Rankin Scale
CEA: Carotid endarterectomy
rScO_2_: Regional cerebral oxygen saturation
BIS: Bispectral index
BP: Blood pressure
Cr: Creatinine
