# Supplementary material for: Risk factors for postoperative acute ischemic stroke in advanced-aged patients with previous stroke undergoing noncardiac surgery: a retrospective cohort study
Source: BMC Surg. 2023 Aug 29;23:258. doi: 10.1186/s12893-023-02162-9 (PMC10466868; doi:10.1186/s12893-023-02162-9)
Supplement: Supplementary file 1 — Additional File 1: Table s1-s3 [file 12893_2023_2162_MOESM1_ESM.docx]

Supplemental Table 1 | The incidence of PAIS with different length of surgery and preoperative mRS

|  | Incidence of PAIS | *P*-value |
| --- | --- | --- |
| Length of surgery |  | <0.001 |
| <3h | 3.1% (9/292) |  |
| ≥3h | 13.9% (15/108) |  |
| Preoperative mRS |  | 0.030 |
| mRS<3 | 5.1% (19/369) |  |
| mRS≥3 | 16.1% (5/31) |  |

Supplemental Table 2 | Multivariate linear Analysis of Perioperative Characteristics and in-hospital expense

| Risk factors | Correlation coefficients(β) | Standard error | *P*-value |
| --- | --- | --- | --- |
| Surgery type | -24959.381 | 4034.885 | <0.001 |
| Length of surgery | 287.673 | 25.437 | <0.001 |
| ASA | 8798.351 | 6910.875 | 0.204 |
| Preoperative mRS | 13657.964 | 10367.108 | 0.188 |

Supplemental Table 3 | Multivariate linear Analysis of Perioperative Characteristics and LOS

| Risk factors | Correlation coefficients(β) | Standard error | *P*-value |
| --- | --- | --- | --- |
| Surgery type | -2.466 | 1.665 | 0.012 |
| Length of surgery | 0.034 | 0.006 | <0.001 |
| ASA | -2.662 | 1.665 | 0.111 |
| Preoperative mRS | 2.159 | 2.497 | 0.388 |
